# Low-Temperature Al-Migration Module for SACM Ge-on-Si Photodiodes

**DOI:** 10.3390/s26217062

**Published:** 2026-11-08

**Authors:** Lovro Marković, Tihomir Knežević, Lis K. Nanver, Ivan Berdalović, Asma Attariabad, Tomislav Suligoj

**Affiliations:** 1 Faculty of Electrical Engineering and Computing, University of Zagreb, 10000 Zagreb, Croatia; 2 Smart Sensors and Microsystems, SINTEF Digital, 0314 Oslo, Norway; 3 Division of Materials Physics, Ruđer Bošković Institute, 10000 Zagreb, Croatia; 4 MESA+ Institute, University of Twente, Hallenweg 23, 7522 NH Enschede, The Netherlands

**Keywords:** Ge-on-Si, APD, SACM, charge layer, Aluminum, material displacement, interface engineering, PureB, shallow junction, material barrier, heterojunction

## Abstract

A low-temperature post-processing Al-migration module is used to achieve separate absorption, charge, and multiplication (SACM) functionality in vertical Ge-on-Si photodiodes without dedicated charge-layer formation by implantation or epitaxy. During a 400 °C alloying step, Al migrates from the contacts through perimeter openings of selectively grown Ge islands and modifies the Ge/Si interface, increasing the conduction-band potential barrier height. This mechanism is supported by *I*–*V* and *C*–*V* characterization and spatially resolved photoresponse under selective illumination of Ge and Si. At low reverse bias, the barrier suppresses carrier collection from Ge, resulting in uniformly low near-infrared (NIR) responsivity. Increasing reverse bias lowers the barrier and increases responsivity before avalanche. Room-temperature optical gain is demonstrated at 1310 and 1550 nm, reaching approximately 5 at 1310 nm. The photoresponse remains spatially uniform across the Ge island at all reverse biases. Light-emission mapping and wavelength-selective laser probing indicate that multiplication occurs predominantly in Si. Separate 1D and 2D TCAD simulations investigate the impact of interface charge and Ge-island geometry on electric-field redistribution, showing that moderate negative interface charge shifts the peak field into Si, while corner-induced field crowding, which will locally lower the interface barrier, facilitates the transport of photogenerated carriers from Ge into high-field regions in Si. The proposed module is potentially compatible with back-end-of-line processing and applicable to other vertical Ge-on-Si structures.

## 1. Introduction

The demand for high-performance near-infrared (NIR) photodetectors is increasing, driven by fiber-optic communications, eye-safe LiDAR systems [1], and on-chip photonics [2]. Detection of low light levels benefits from internal gain, and high-gain photodetectors are also being developed in emerging material platforms [3,4]. Nevertheless, considerable effort is still devoted to Ge-on-Si avalanche photodiodes (APDs), owing to their compatibility with Si photonics and suitability for large-scale integration [5,6,7,8,9,10,11,12]. Integrated Ge-on-Si platforms open possibilities for device structures with separate absorption, charge, and multiplication (SACM) regions, in which Ge acts as the NIR absorber while avalanche multiplication is confined to Si, thereby minimizing dark current and excess noise [7]. This generally requires a highly doped charge layer to control the field distribution between the absorption and multiplication regions. The layer is typically formed by epitaxial growth of a lightly doped Si buffer layer between the Ge absorber and the substrate [10,11,12,13,14], followed by ion implantation and high-temperature annealing [11,12,13,14,15]. SACM Ge-on-Si APDs without Si epitaxy have been demonstrated [15], but still require ion implantation and mesa etching. Moreover, the introduced highly doped charge layer necessarily creates a potential barrier at the Ge/Si heterointerface. If the barrier is not sufficiently lowered before avalanche onset, carrier transport from Ge to the multiplication region is suppressed, reducing the detection efficiency [8,12,14].

**Figure 1:**
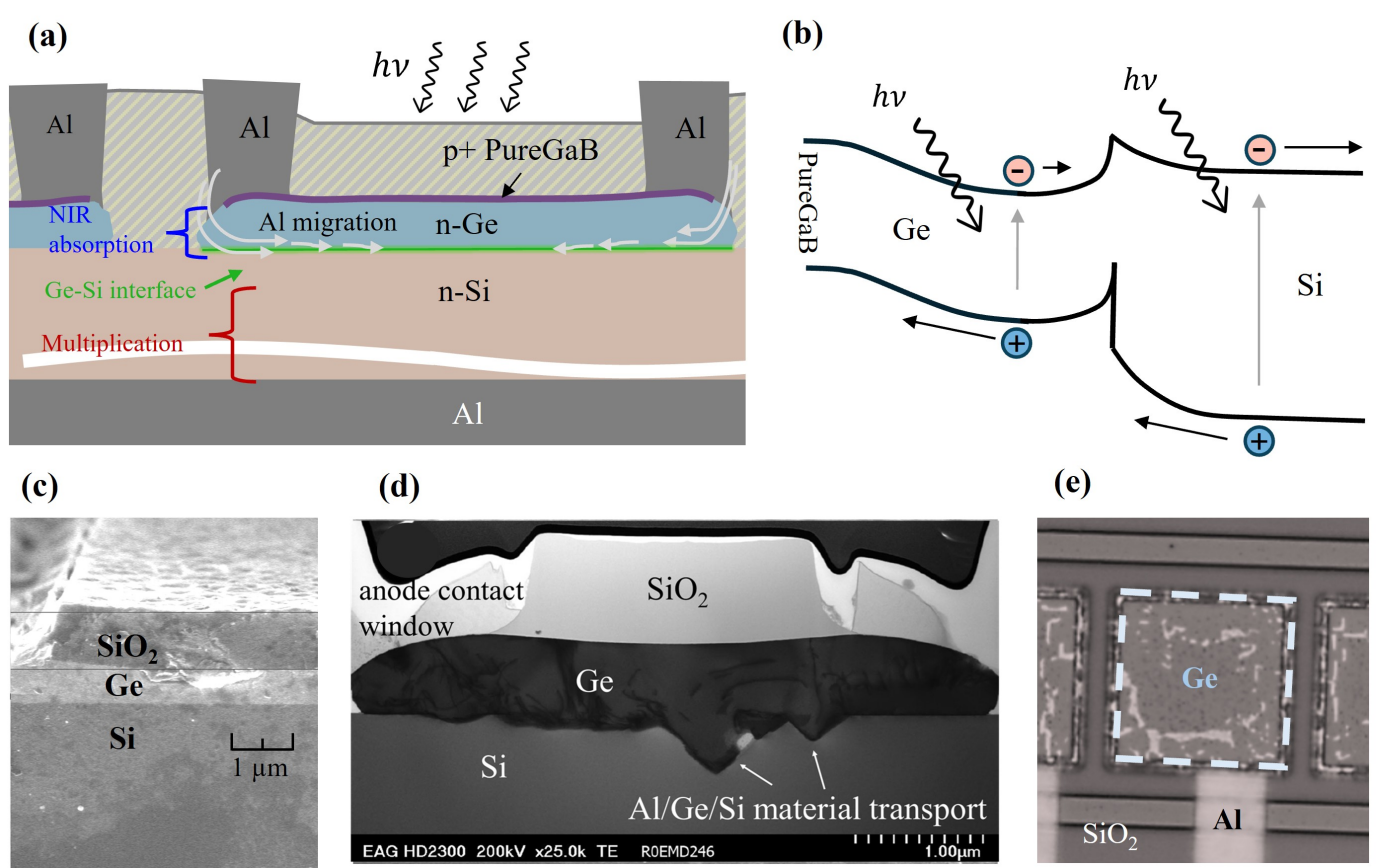
(**a**) Schematic cross-section of PureGaB diodes and the Al migration during alloying. SACM functional regions (absorption, charge, and multiplication) are indicated. (**b**) Schematic band diagram. (**c**) SEM image of a cross-section of a 26 × 26 µm^2^ diode, showing Si/Ge/PECVD SiO_2_ material layers. Ge loading is observed within ∼2 μm from the edges. (**d**) TEM image of a 5 × 5 µm^2^ Ge island after Al migration and material displacement at the Ge/Si interface. (**e**) High-resolution optical image of a fully fabricated PureGaB diode.

Selective Ge epitaxy in oxide windows on Si enables monolithic integration of Ge absorption and Si multiplication regions, with threading dislocation density below 10^7^ cm^−2^ [16]. Combined with ultrashallow junctions formed by nanometer-thin boron (PureB) or gallium–boron (PureGaB) deposition without introducing process-induced defects, this approach has been used to fabricate monolithic p(i)n Ge-on-Si photodiodes with a broadband UV-to-NIR response [17,18]. A typical PureGaB Ge-on-Si photodiode is shown in Figure 1a. During metallization, PureGaB (PureB) prevents reactions between Al and the underlying semiconductor [19,20]. However, where Ge remains exposed at the device perimeter, Al can migrate along the Ge sidewalls to the Ge/Si interface and then spread laterally along the interface during contact alloying, which is typically performed at 400 °C [18,19,21]. Previous studies [19,21,22] identified Al grains at the Ge/Si interface and local redistribution of Ge and Si associated with Al-mediated material transport. Cross-sectional TEM/HRTEM revealed Si pitting and Ge-filled V-shaped grooves extending up to 5 μm from the device perimeter [19]. These modifications were absent where Ge was protected by PureGaB, directly relating the observed material redistribution to the impact of Al migration. EDS measurements confirmed the presence of Al grains at the interface but could not resolve possible Al doping of the surrounding Ge [19].

The localized nature of Al-mediated material transport significantly limited possibilities for structural and compositional analysis. Subsequent studies therefore focused on electrical and optical characterization with respect to different alloying temperatures and durations [18,21,22]. Al migration increased strongly with both alloying temperature and duration. At 500 °C, extensive migration even caused visible consumption of Al contact pads connected to exposed Ge/Si perimeter gaps [22]. Prolonged alloying also progressively reduced zero-bias responsivity, which was initially attributed to increased carrier trapping. However, responsivity recovered with increasing reverse bias to values comparable to those of devices subjected to shorter alloying times, which generally exhibited higher responsivity and weaker bias dependence [21]. Reverse leakage current also decreased with prolonged alloying [21,23]. These observations suggested that an electrostatic barrier, rather than increased recombination, was the dominant cause of reduced responsivity [21], as illustrated by the band diagram in Figure 1b. Further evidence was provided by temperature-dependent broadband responsivity measurements (900–1600 nm) [21], which showed a stronger temperature dependence for Ge-generated photocurrent than for Si-generated photocurrent. This behavior is consistent with a potential barrier/well at the Ge/Si interface that attenuates carrier transport of, respectively, electrons/holes to the cathode/anode [21].

In this work, the low-temperature Al-migration module is used to achieve SACM functionality in a Ge-on-Si photodiode. The previously proposed interface-barrier model is further investigated through spatially resolved optical measurements and *C*–*V* and *I*–*V* characterization. Avalanche operation is demonstrated, with NIR optical gain measured at both 1310 and 1550 nm. The location of maximum multiplication is investigated by selective laser probing at different wavelengths. 1D and 2D TCAD simulations are used to evaluate the impact of interface charge on the electric-field distribution between the Ge and Si.

## 2. Fabrication with Al-Migration and Measurement Techniques

Device fabrication starts with plasma etching of windows in an isolation oxide on 2–5 Ω·cm (100) n-Si wafers, uniformly phosphorus-doped to ∼10^15^ cm^−3^. Selective Ge epitaxy is then performed in a commercial CVD reactor, beginning with a seed layer grown at 400 °C, followed by As-doped Ge growth at 700 °C (∼10^16^ cm^−3^), and a 60 min anneal at 750 °C. In the same reactor, the p^+^ anode is subsequently formed by depositing a Ga wetting layer followed by a B layer (PureGaB), with a total thickness of 11 nm. The wafer was then capped with a 1-μm-thick PECVD oxide. Cross-sectional scanning electron microscopy (SEM) revealed a ∼500 nm thick Ge layer, with loading effects within 2 μm of the island edges, and a uniform 1 μm thick passivation oxide (Figure 1c). Anode contact windows were etched through the PECVD oxide down to the PureGaB layer along the Ge perimeter, followed by Al deposition and patterning. PureGaB generally prevents Al–Ge reactions [19,24]. However, slight oxide over-etching exposed approximately 100 nm of the Ge island edge that was not covered by PureGaB, since it had been protected by the Ge-window sidewall oxide during PureGaB deposition. During the final 400 °C, 60 min alloying step, these exposed regions enabled Al migration along the Ge island sidewalls and further laterally along the Ge/Si interface, as investigated in more detail in [19,21,22]. The resulting interface modification, marked by the formation of Ge-filled V-shaped grooves characteristic of this process, was also observed in the present devices (Figure 1d). The n-Ge/Si cathode was back-contacted by Al. A top view of the fabricated device is shown in Figure 1e.

Optical characterization was performed using pigtailed laser diodes at 638, 1310, and 1550 nm. Calibrated room-temperature responsivity and temperature-dependent measurements were performed on PCB-bonded devices using a laser beam exceeding 40 μm in diameter to illuminate the entire Ge island. Spatially resolved laser probing was performed to examine the lateral distribution of the optical response associated with the interface modification. For this purpose, devices were wire-bonded to a PCB and mounted on a 3D translation stage with sub-μm resolution. A high-NA, long-working-distance lens provided minimum FWHM spot sizes of 1.7 μm at 638 nm and 2.6 μm at 1310 nm.

## 3. Optoelectronic Characterization

**Figure 2:**
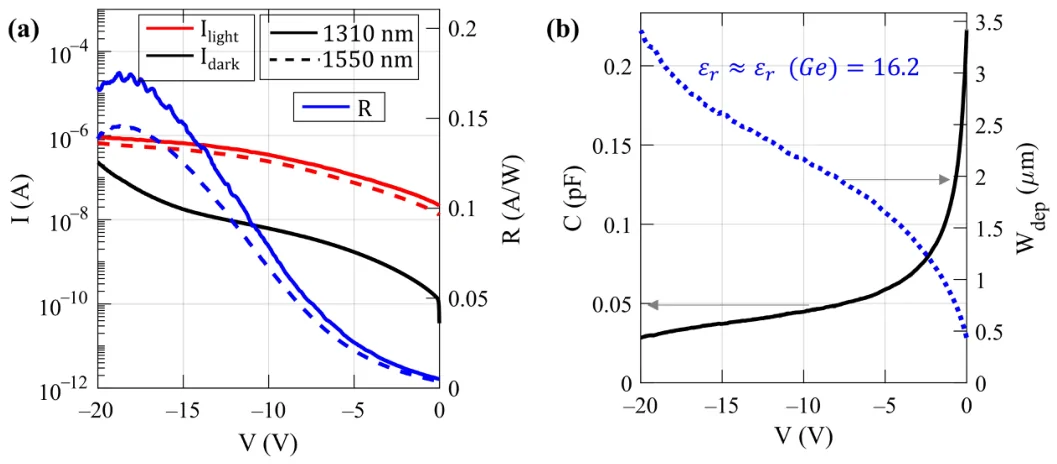
Electrical characterization of PureGaB diodes, showing (**a**) dark current, photocurrent, and focused-laser responsivity (black, red, and blue lines, respectively) at 1310 nm (solid) and 1550 nm (dashed). (**b**) Diode capacitance after de-embedding pad parasitics (solid) and estimated depletion width (dashed). The arrows in (**b**) indicate the vertical axis corresponding to each dataset. The depletion width was estimated from the capacitance using the parallel-plate capacitor approximation with the relative permittivity of Ge ($\varepsilon_{r}=16.2$).

Focused responsivity measurements were first performed from 0 to 20 V reverse bias at wavelengths of 1310 and 1550 nm, as shown in Figure 2a. The responsivity was calculated as $R=\left(I_{\mathrm{light}}-I_{\mathrm{dark}}\right)/P_{\mathrm{in}}$, where $P_{\mathrm{in}}$ is the incident optical power. Since both wavelengths are absorbed exclusively in Ge, the measured photocurrent directly probes carrier collection from the Ge absorber. The zero-bias responsivity is 4 and 3 mA/W at 1310 and 1550 nm, respectively. For comparison, devices from our previous fabrication run, subjected to only a 30 min contact-annealing step, exhibited an average zero-bias responsivity of about 60 mA/W [18,21]. However, the present devices show a much stronger increase in responsivity with reverse bias, reaching values comparable to those of the 30 min annealed devices at about 15 V [21]. With a further increase in reverse bias, the responsivity reaches 176 and 146 mA/W at 1310 and 1550 nm, respectively, at 19 V.

To determine whether this increase originates from progressive depletion of the Ge absorber, *C*–*V* measurements were used to estimate the depletion width. The measurements were performed up to 20 V reverse bias, with the upper limit imposed by the increasing leakage current, and are shown in Figure 2b. The extracted capacitance indicates that the 500 nm-thick Ge absorber is already fully depleted near zero bias. Using a parallel-plate-capacitor approximation, the estimated depletion width exceeds the Ge thickness at approximately 0.2 V reverse bias. The dielectric constant of Ge was used. Correctly describing the subsequent extension of the depletion region into Si would additionally require accounting for the dielectric constant of Si. The complete depletion of Ge near zero bias demonstrates that the increase in NIR responsivity cannot be attributed to progressive depletion of the absorber. Instead, it indicates that carrier collection is limited by transport across the Ge/Si interface.

The *C*–*V* characteristics also exhibit an inflection at approximately 15 V, accompanied by a more rapid increase in the extracted depletion width at higher biases. This behavior is consistent with substantial lowering of the Ge/Si interface barrier and increased coupling between the depleted Ge and Si regions. The inflection occurs within the same bias range in which the responsivity approaches the values measured in devices subjected to the shorter annealing step, further supporting the interface-barrier model.

**Figure 3:**
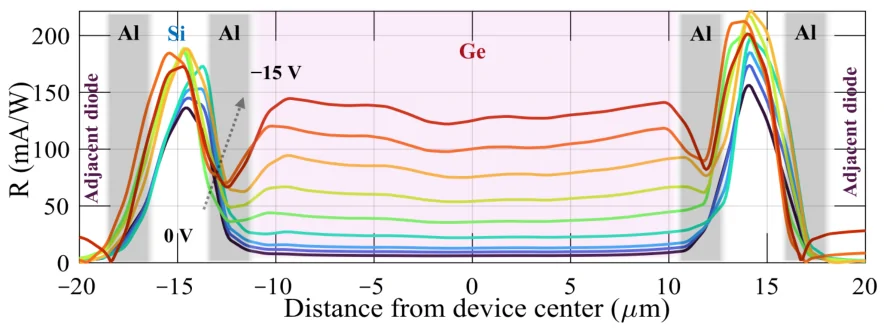
Focused line-scan responsivity profiles of the PureGaB Ge-on-Si photodiode measured at 638 nm for reverse biases from 0 to 15 V. The curves are shown using a continuous color sequence from purple to red, corresponding to increasing reverse bias and responsivity at the center of the Ge island (X = 0). The positions of the Ge island, Al metallization tracks, and openings exposing the Si substrate are indicated.

To further assess the interface-barrier model, spatially resolved line-scan measurements were performed. The oxide openings surrounding the Ge islands expose the Si substrate between neighboring devices in the array (see Figure 1a). This geometry enables selective illumination of the Ge absorber and the underlying Si substrate through the narrow opening at the oxide perimeter, as shown in Figure 3. At low reverse bias, illumination of Ge produces a low photocurrent, whereas illumination of Si yields a stronger response. The latter behavior is consistent with electrons (photo)generated in Si not being affected by the interface barrier in their drift from the absorption site to the respective electrode, as illustrated in Figure 1b. With increasing reverse bias, the responsivity increases for illumination of both regions, but the increase is considerably stronger for Ge illumination, as expected from progressive suppression of the interface barrier. The relatively smaller increase measured under direct Si illumination can be attributed to the widening of the depletion region in Si. At approximately 15 V, corresponding to the inflection in the *C*–*V* characteristics, the response from Ge reaches that from Si, indicating complete barrier lowering.

In addition to separating the responses from Ge and Si, the same line-scan profiles reveal the spatial uniformity of the bias-dependent response. At low bias, the responsivity is uniformly low across the Ge island, with no localized regions of higher response. The slightly higher response near the device edges may originate from variations in the Ge absorber thickness or from the shorter distance to the device corners, where electric-field crowding occurs, as discussed later. As the reverse bias increases, the responsivity rises across the full scanned width. This indicates that the suppression of carrier collection associated with prolonged Al alloying, and its subsequent recovery with reverse bias, are not exclusively confined to the regions where Al-mediated displacement of Ge and Si was directly observed by TEM.

**Figure 4:**
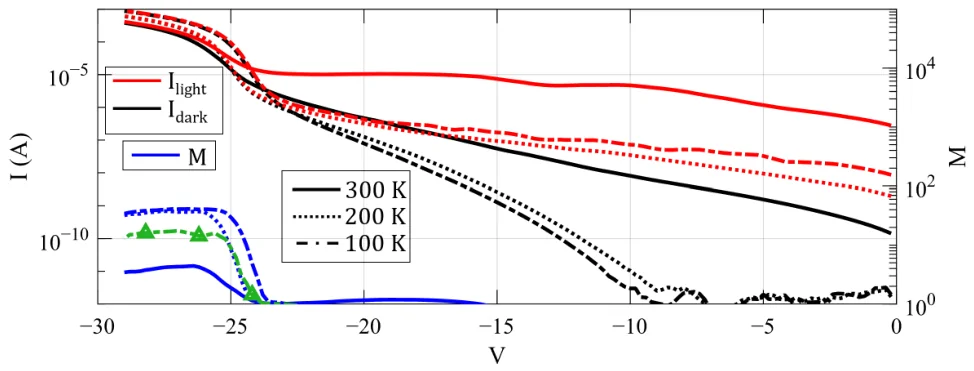
Dark current, photocurrent, and optical gain (M) under 1310 nm illumination in the avalanche regime. *M* is calculated according to Equation (1). The measurements were performed at different temperatures (300 K, 200 K, and 100 K). For 1550 nm illumination, the profile with the highest gain (100 K) is shown (green).

The *I*–*V* characteristics were then acquired up to breakdown to evaluate the avalanche response. The devices enter avalanche multiplication gradually, with the breakdown voltage determined from the maximum derivative of the reverse current, resulting in ∼24 V. The dark current, photocurrent, and optical gain are shown in Figure 4. Reverse biases up to 50 V caused no device degradation, while the maximum current in breakdown is limited by the series resistance. The avalanche response is gradual rather than abrupt, resulting in a continuous increase in carrier multiplication with reverse bias until the current is ultimately limited by the series resistance. Consequently, the device current remains well controlled throughout the multiplication regime and can be reproducibly switched between the dark and illuminated states, even at the breakdown voltage. Optical gain was calculated for each dataset as

(1) $$M=\frac{I_{\mathrm{light}}\left(V\right)-I_{\mathrm{dark}}\left(V\right)}{I_{\mathrm{light}}\left(V_{0}\right)-I_{\mathrm{dark}}\left(V_{0}\right)},$$

where the numerator and denominator represent the photogenerated current, defined as $I_{\mathrm{light}}-I_{\mathrm{dark}}$, thereby excluding the dark-current contribution. Since the barrier-limited responsivity increases with reverse bias before the onset of multiplication, $V_{0}$ cannot be selected at the point of full Ge absorber depletion, as typically done. After lowering the conduction-band barrier at around 15 V, the photocurrent increases negligibly up to the breakdown voltage (around 24 V), indicating that avalanche multiplication does not start in this voltage range. Therefore, V0 is set to 23 V, just before the onset of hard breakdown. The same value of V0 is used for gain extraction at 200 K and 100 K since the breakdown voltage does not change significantly with temperature.

At room temperature, the gain at 1310 nm reaches 4.4 and is limited by the series resistance in breakdown. Operation at 1550 nm was also demonstrated, with similar gain characteristics observed among devices on the same die. Upon cooling, the extracted optical gain increases. The cooled devices exhibit higher optical gain than at room temperature, reaching 38.0 and 41.1 at 200 and 100 K, respectively. This increase is primarily due to the higher currents reached at breakdown before the series resistance becomes limiting.

**Figure 5:**
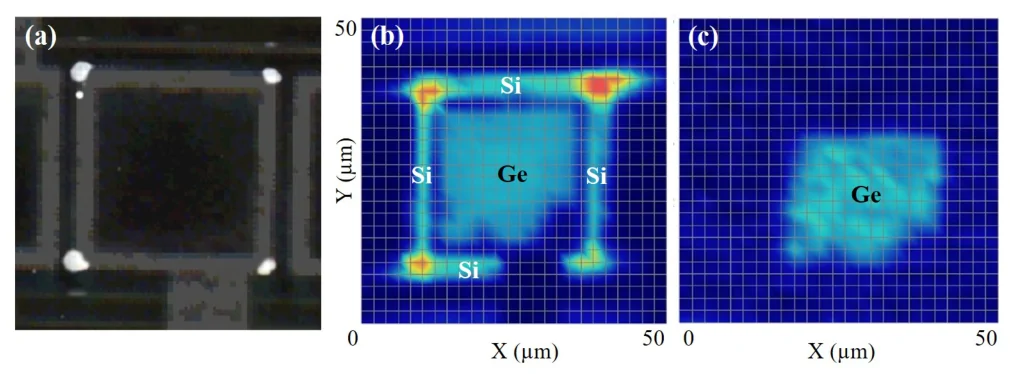
Diodes biased beyond breakdown (27 V reverse bias), showing (**a**) an optical microscopy image of light emission from a PureGaB diode with the site of maximum emission seen in the Si under the oxide, and 2D responsivity maps for (**b**) 638 nm and (**c**) 1310 nm.

A light-emission test was performed on devices in breakdown to identify the sites of peak avalanche [25] (Figure 5a). The maximum emission intensity from the diode is located at the corners, outside the Ge perimeter metallization, indicating breakdown in Si beneath the passivation oxide. This is further supported by focused responsivity profiling at 638 nm, absorbed in both Si and Ge, and 1310 nm, absorbed exclusively in Ge, performed here at 27 V reverse bias, beyond breakdown. The beams were scanned across the diode in 2 µm steps, and the photocurrent was recorded at each position. Consistent with the low-to-moderate-bias uniformity discussed above, the optical response was uniform across the Ge islands at both wavelengths. However, at 638 nm, an additional high-responsivity ring was observed around the device perimeter, originating from direct illumination of Si through the non-metallized oxide spacing between the adjacent devices. The low-response region between the Ge island and the surrounding Si is attributed to shading by the Al contact ring and tracks (Figure 1). When focusing 638 nm to the corners, a strong photocurrent peak was observed (Figure 5b). In contrast, focusing 1310 nm, which is not absorbed in Si, to the same spots yielded negligible photocurrents (Figure 5c), indicating that the corner emission peak, as well as the enhanced corner response to 638 nm illumination, originates from carrier multiplication in Si.

The corner localization of the emission and of the peak photoresponse is attributed to electric-field crowding at the device corners, an inherent consequence of the rectangular device layout. This locally enhanced field lowers the Ge/Si interface barrier at the corners, channeling photogenerated electrons from Ge into the Si multiplication region and favoring breakdown initiation there. This could be related to slightly higher response observed near the device edges, as previously noted with respect to Figure 3.

## 4. TCAD Simulations

The observation of optical gain at NIR wavelengths, for which absorption occurs in Ge, with multiplication occurring predominantly in Si, demonstrates the separation of the absorption and multiplication regions. The Al-induced interface barrier is expected to govern both carrier transport across the Ge/Si interface and the redistribution of the electric field between the Ge and Si regions. To examine both effects qualitatively, the interface barrier was modeled in TCAD as an effective charge, uniformly distributed along the Ge/Si interface of a simplified one-dimensional PureGaB/Ge/Si stack. The model followed the PureGaB process simulations reported in [26] and used the nominal geometry of the fabricated devices. The PureGaB layer was represented by a Gaussian p-type profile with a peak concentration of $10^{20}$ cm^−3^ and a junction depth of 100 nm, and the Ge and Si regions were uniformly n-type doped to $10^{16}$ and $10^{15}$ cm^−3^, respectively. The density and microscopic origin of the Al-induced interface charge have not been determined experimentally; therefore, three representative charge densities were simulated.

**Figure 6:**
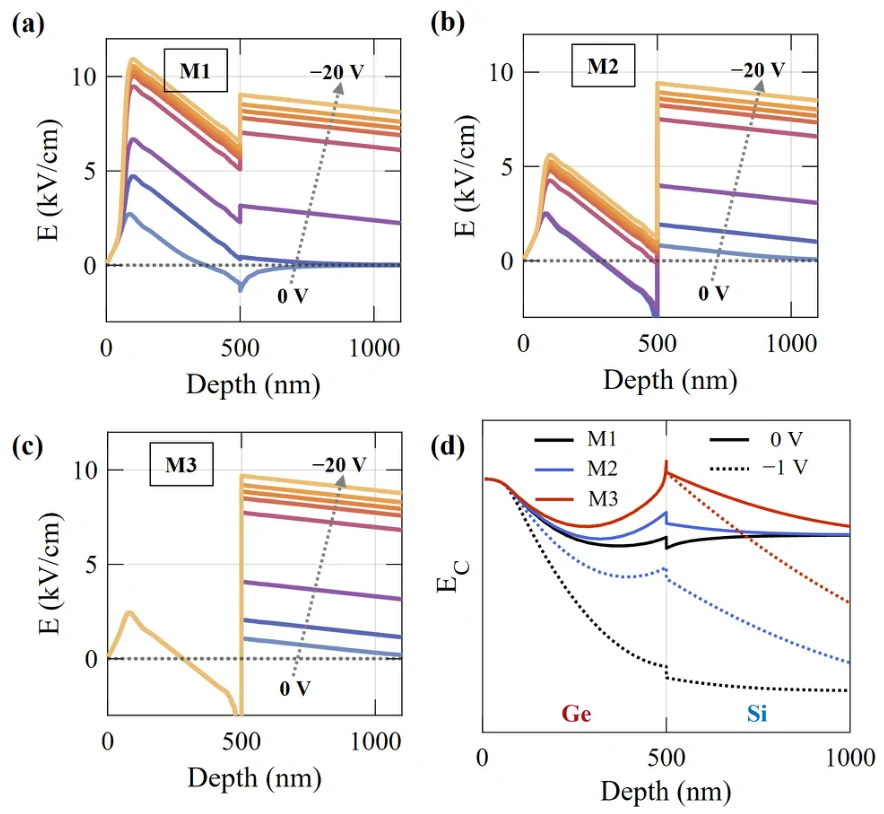
Simulated 1D TCAD electric-field profiles at reverse biases of 0, 1, 2, 5, 10, and 20 V for (**a**) no interface charge (M1), (**b**) a negative sheet-charge density of $3\times 10^{11}$ cm^−2^ (M2), and (**c**) $5\times 10^{12}$ cm^−2^ (M3) at the Ge/Si interface. The curves are shown using a continuous color sequence from blue to orange, corresponding to increasing reverse bias from 0 to 20 V and the resulting increase in the electric field, as indicated by the arrow. The charge densities in M2 and M3 were selected as illustrative values to represent two distinct regimes of electric-field redistribution with increasing reverse bias. The positive field direction is defined from the bottom (cathode) to the top (anode) contact. (**d**) Conduction-band profiles at 0 and 1 V reverse bias for M1–M3.

Figure 6 shows the simulated electric-field and conduction-band profiles across the Ge/Si stack under increasing reverse bias for three cases: no interface charge (M1), moderate interface charge (M2), and excessive interface charge (M3). Since the one-dimensional model neglects field crowding at the device corners, where avalanche breakdown is expected to be initiated, the simulated fields underestimate the local peak fields in the fabricated devices. The model is therefore used to identify, for each case, where the field peaks in the vertical direction and how the interface barrier responds to reverse bias, rather than to predict breakdown voltages.

For the diode without interface charge at the Ge/Si interface (M1), the electric-field maximum at zero bias is located at the p–n junction in Ge. With increasing reverse bias, the applied potential drops across the Ge layer, as indicated by the conduction-band evolution with bias (Figure 6d), increasing the electric field and eventually leading to breakdown at the p–n junction in Ge.

With moderate negative (acceptor-like) interface charge levels (as represented by M2), the externally applied bias is distributed partly across Ge and partly across Si, reducing the field in Ge. The field maximum shifts to Si, leading to breakdown in Si near the heterointerface. However, as compared to M1, a conduction-band barrier forms at the Ge/Si interface (Figure 6d), appearing as a negative electric-field component at the bottom of the Ge layer (Figure 6b). At low reverse bias, this barrier blocks electrons generated in Ge from reaching the cathode and confines photogenerated holes near interface defects, enhancing their recombination. Both mechanisms contribute to the reduced low-bias responsivity. Assuming temperature-independent interface charge, with the barrier dominating electron transport across the heterointerface, the electron current across the interface is governed by thermionic emission [27,28,29]:

(2) $$I_{e,\mathrm{int}}\left(V_{\mathrm{ext}},N_{\mathrm{int}},T\right)\propto \exp\left(-\frac{\Phi (V_{\mathrm{ext}},N_{\mathrm{int}})}{k_{B}T}\right),$$

where the effective conduction-band barrier height (Φ) depends on the interface charge ($N_{\mathrm{int}}$) and the applied reverse bias ($V_{\mathrm{ext}}$). Increasing the reverse bias reduces the negative electric-field component and, consequently, the interface barrier Φ. This enhances electron emission across the Ge/Si interface, thus reducing the probability that electrons recombine in the anode region. Moreover, the trapping of holes in the highly defective potential well at the Ge/Si interface is reduced, and the responsivity is increased before the onset of multiplication, as observed in our devices.

In the case of excessive interface charge (M3), externally applied bias drops exclusively across Si, while the field and potential distribution in Ge remain constant, preventing the interface barrier from being lowered by the applied bias. Although such a charge layer ensures multiplication in Si (with the electric-field peak located in Si), the interface barrier blocks photogenerated carrier transport across the heterointerface, i.e., from the absorption to the multiplication region. Carriers generated in Ge recombine before reaching the multiplication region, leading to the loss of detection efficiency. The M3 case thus manifests as low quantum efficiency and low, bias-independent responsivity before breakdown.

Of the three cases, only M2 is qualitatively consistent with the measured characteristics. M1 places the field maximum, and thus multiplication, in Ge, which contradicts the observed multiplication predominantly in Si. M3 suppresses the NIR responsivity at all biases below breakdown, which contradicts the observed increase in responsivity with reverse bias and the NIR optical gain. In M2, the peak field is confined to Si, while the bias-dependent barrier allows transport of photogenerated electrons from the Ge absorber into the Si multiplication region as the reverse bias increases. The Al-induced potential barrier could be modeled as an interface charge layer, enabling SACM operation with NIR absorption in Ge and avalanche multiplication in Si.

**Figure 7:**
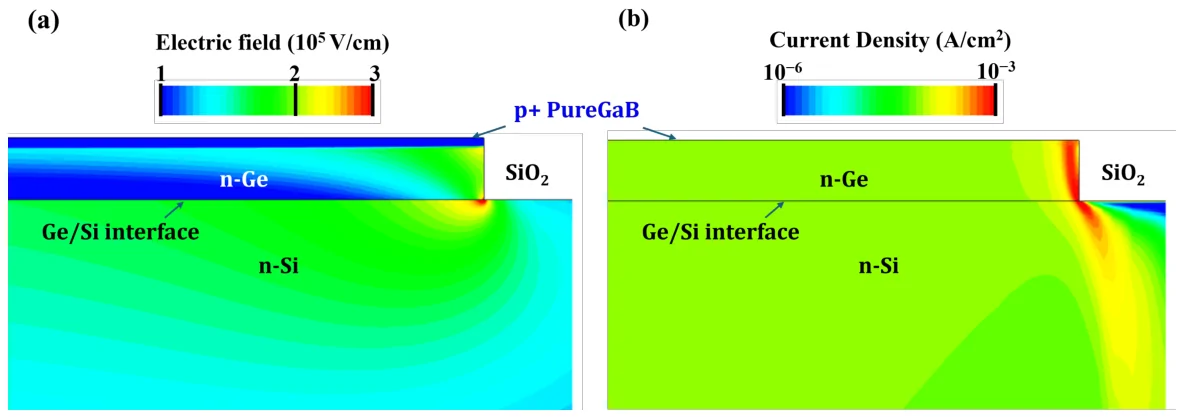
Simulated 2D TCAD distributions of (**a**) the electric field and (**b**) the current density at breakdown for a uniform negative fixed-charge density of $3\times 10^{11}$ cm^−2^ along the Ge/Si interface.

To further assess the impact of Ge-island corners, 2D simulations were performed as an extension of the 1D model using the fabricated geometry, comprising a 26 µm-wide Ge island within oxide windows on a 500 µm-thick Si substrate. The doping profiles were identical to those used in the 1D simulations, without lateral variations. A uniform negative fixed charge density of $3\times 10^{11}$ cm^−2^ was used at the Ge/Si interface, which was shown by 1D simulations to provide the most appropriate interpretation of the measurements. At the photodiode reverse bias, the depletion region spreads into the silicon below the Ge island and also laterally below the isolation SiO_2_. Such high positive charge on the Si side of the interface causes the electric field to increase at the Si/Ge/oxide corner, as can be seen in the electric field distribution in Figure 7a. The local increase in the electric field implies a high potential drop, which causes the conduction-band barrier to decrease, resulting in a higher local current density, as observed in light emission measurements and the responsivity map in Figure 5. The spatial charge distribution as a result of the Ge-island geometry determines the preferential current paths at Ge-island edges (Figure 7b). In the actual device, the effect is enhanced further by the top-view device corners, but is also affected by the Ge-island sidewall surface passivation, oxide charge, and other process parameters.

In summary, both 1D and 2D simulations indicate that an intermediate interfacial barrier is required to balance electric-field localization and carrier collection from the Ge absorber. This interpretation is consistent with the measured increase in NIR responsivity up to approximately 15 V and the predominantly Si-based multiplication observed in the fabricated devices. Due to the electric field distribution, the corner effects provide a path for photogenerated carriers to reach the Si multiplication region directly from Ge.

## 5. Conclusions

SACM operation was demonstrated in PureGaB Ge-on-Si photodiodes using a low-temperature Al-migration module introduced during a 400 °C, 60 min contact-alloying step. The measured behavior is consistent with the formation of an Al-induced potential barrier at the Ge/Si interface, which controls carrier transport across the heterointerface. Increasing the reverse bias lowers this barrier, resulting in a uniform recovery of carrier collection across the Ge island and enabling carrier transfer from the Ge absorption region to the Si multiplication region at approximately 15 V, before the onset of avalanche multiplication. TCAD simulations attribute SACM operation to a moderate conduction-band barrier associated with negative interface charge, which shifts the peak electric field into Si and effectively acts as a charge layer. Corner-induced barrier lowering may further facilitate carrier transport into localized high-field regions in Si, directing carriers toward the multiplication region. NIR optical gain was demonstrated at both 1310 and 1550 nm, reaching 4.4 at 1310 nm at room temperature, with absorption in Ge followed by multiplication predominantly in Si. Since the interface is modified within the existing contact-alloying step, without Si epitaxy, ion implantation, mesa etching, or additional high-temperature processing, the presented process module offers the possibility of CMOS back-end-of-line integration and is readily applicable to other vertical Ge-on-Si structures.

## Data Availability

Dataset available on request from the authors.

## Abbreviations

The following abbreviations are used in this manuscript:

APD	Avalanche photodiode
CMOS	Complementary metal–oxide–semiconductor
CVD	Chemical vapor deposition
EDS	Energy-dispersive X-Ray spectroscopy
FWHM	Full width at half maximum
HRTEM	High-resolution transmission electron microscopy
NIR	Near-infrared
PECVD	Plasma-enhanced chemical vapor deposition
SACM	Separate absorption, charge, and multiplication
SEM	Scanning electron microscopy
TCAD	Technology computer-aided design
TEM	Transmission electron microscopy
